# Biological Effects of Medicinal Plants on Induced Periodontitis: A Systematic Review

**DOI:** 10.1155/2016/3719879

**Published:** 2016-09-21

**Authors:** Jefferson Soares de Oliveira, Moara e Silva Conceição Pinto, Lucas de Araújo de Bastos Santana, Antonione Santos Bezerra Pinto, David di Lenardo, Daniel Fernando Pereira Vasconcelos

**Affiliations:** ^1^Laboratory of Biology and Biochemistry Plants (BIOqPLANT), Federal University of Piaui, Parnaiba, PI, Brazil; ^2^Laboratory of Histological Analysis and Prepare (LAPHIS), Federal University of Piaui, Parnaiba, PI, Brazil; ^3^Department of Morphology, LABICONTE, Federal University of Ceara, Fortaleza, CE, Brazil

## Abstract

*Objective*. The aim of this systematic review was to investigate the advances in the study of medicinal plants and their biologic effects on periodontitis in animal models.* Study Design*. A systematic search was conducted by three independent researchers, who screened articles published up to March/2016, to identify the studies that contained sufficient and clear information on the association of the medicinal plants and periodontitis in murine models. The searches were performed using PubMed, Cochrane, and Science Direct databases*. Results*. After a critical analysis of titles and abstracts, 30 studies were finally eligible for analysis. The studies presented a great diversity of the experiment designed regarding the methods of induced periodontitis and the evaluation of the medicinal plants efficacy. None of the studies described the possible toxic effects associated with the administration of the plant material to animals and whether they could prevent damage to organs caused by systemic effect of induced periodontitis. Gel-based formulations containing plant substances are seen as an interesting strategy to treat periodontitis.* Conclusions*. In this systematic review, the state-of-the-art knowledge on the medicinal plants and the induced periodontitis was critically evaluated and discussed from the experiment designed to the possible clinical application.

## 1. Introduction

Periodontitis is one of the most extensive oral problems that affect human population, resulting from an inflammatory response against microorganisms involved with plaque accumulation on the subgingival dental surface. The development of the process depends on the interaction between the bacteria in the site of infection. The presence of an oral biofilm composed by bacteria and their products includes also lipopolysaccharides and proteinases that are responsible for the progression of periodontitis. Bacteria stimulate host immunopathological and inflammatory mechanisms that result in the destruction of the periodontal tissue [1].

Different strategies have been used to treat periodontal diseases. Mechanical therapy and surgical procedures reduce microbial burden, being effective in the control of the periodontitis progression. Nevertheless, this regulation is not always satisfactory, possibly due to the prominent role of immunogenetic response on periodontal destruction. In some cases, adjunctive therapies may be required [2]. Thus, the discovery and development of potential therapeutic drugs with the ability to regulate the host immune and bacteria-mediated inflammatory interactions are a valuable approach for the prevention and treatment of the periodontal disease [2–4]. In this context, plants can represent an interesting source of molecules with a potential activity against periodontal disease progression. The growing incidence of periodontitis, the increased resistance of oral bacteria to antibiotics, and the adverse effects of some drugs used in dentistry all motivate the search for safe and effective molecules to treat and prevent the disease [5].

Medicinal plants have been fundamental for thousands of years to provide bioactive molecules used to treat different types of human infirmities, such as inflammation, pain, and tumors. Also, they can be a source of compounds to be tested in the treatment of periodontal diseases. Nowadays, there is an increasing number of scientific investigation exploring plant extracts or purified molecules in periodontal diseases [3]. Several of these studies were performed using animal models since biochemical, histological, and anatomical features are similar to humans [3, 4, 6, 7].

The aim of this systematic review was to investigate the advances in the study of medicinal plants and the development of induced periodontitis in animal models. Data were described and discussed in order to evaluate the limitation and also the perspectives of the application of these agents in the treatment of the periodontal disease.

## 2. Material and Methods

**Figure 1:**
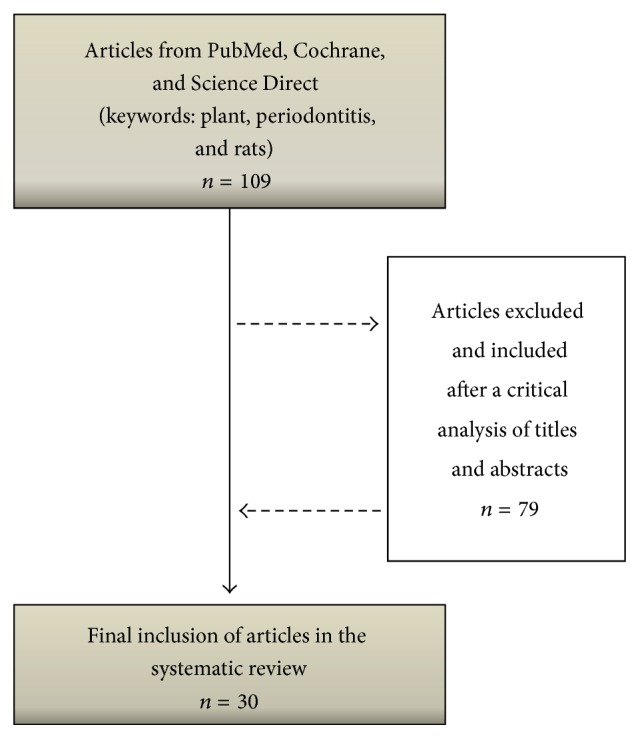
Search flowchart and selection of articles for the review of the literature.

A systematic search was conducted by three independent researchers who screened articles published up to March/2016 in order to identify the studies that contained sufficient and clear information on the association of the medicinal plants and periodontitis in murine models. The searches were performed using PubMed (http://www.ncbi.nlm.nih.gov/pubmed), Cochrane (http://www.cochranelibrary.com/), and Science Direct (http://www.sciencedirect.com/) databases using the key words* plant*,* periodontitis,* and* rats* and search details “plant” [All Fields] AND “periodontitis” [All Fields] AND “rats” [All Fields]. The articles selected by each researcher were compared to remove duplicate records and 109 different articles were initially evaluated. After a critical analysis of titles and abstracts, 79 articles were excluded such as articles that were not in English, articles that were not fully available, or those that did not report an association between medicinal products and murine model of periodontitis. Hence, 30 studies were finally eligible for a qualitative analysis for this review (Figure 1).

## 3. Results and Discussion

Table 1 summarize plant species, plant material, route of administration, animal used, type of induction and time of analysis, and the ability of the medicinal plant to reduce alveolar bone loss related to 30 selected articles.

The majority of articles (43.3%) described studies conducted with plant extracts, 40% with purified compounds and 16.6% with a mixture of two or more plant extracts. Among them, six articles described experiments developed using a commercial product [8–13], containing purified compounds or a mixture of plant extracts. None of authors mentioned the process of obtaining selected plant material as an important limitation for development of the research.

In general, studies were preferentially conducted using* Rattus norvegicus* (90%) of different strains Wistar, Lewis, or Sprague-Dawley. This specie is one of the mostly used for* in vivo* experimental models, including the pathogenesis of periodontal disease because they offer some advantages such as price, easy handling, and the possibility of microbiological, macroscopic, and histological evaluation [14]. Other studies were conducted using* Mus musculus* Balb/c (6.6%) or C57BL/6. Rodents were preferred as the animal model since they present biochemical, histological, and anatomical features similar to humans [14].

Taking into consideration the sex of the animal, most authors chose to use males (86.6%). Two articles described the use of females [12, 15] and two studies were conducted using males and females [4, 8]. One study did not describe the sex of the animals [16]. Most* in vivo* studies which investigate the biological effect of plant substances favor the use of male animals. This choice is based on the fact that female hormones should interfere in the development and progression of the disease [17]. None of the articles analyzed was related to the hormonal influence on the biological activities investigated. However, studies conducted with females could clarify the effect of their hormones on the specific pathways involved in the periodontitis progression.

Regarding the common type of periodontitis-induction, in 66.6% of the articles, the process was caused by ligature (silk: 23.3%, nylon: 23.3%, and cotton: 20%). This method is widely used because it facilitates the accumulation of biofilm. This procedure increases the infiltration of inflammatory cells and the production of chemical mediators that lead to the degradation of the tissues around the teeth contributing to the destruction of the periodontal tissues [1]. In addition to periodontitis induced by ligature, 16.6% of articles described the gingival injection of bacteria, such as* P. gingivalis *and 16.6% described the administration of* E. coli* endotoxin (LPS) or* S. griseus* (proteases) to promote the periodontal disease. The inoculation of bacteria or their subproducts leads to an inflammatory response different from that promoted by periodontitis induced through induction with ligature [18].

Some of the studies analyzed described that long periods of experimental design were utilized to properly investigate the severity of tissue destruction during periodontal disease treatment: 63 days [8]; 56 days [19]; 42 days [20]; and 50 days [21]. However, the time of analysis changed substantially. More than 55% of the studies performed experiments during one to two weeks, 26.6% for three to four weeks, and 13.3% during six weeks or more. One study was conducted with an acute periodontitis rat model in 24 hours [22]. These variations in the experimental periods may be due to factors such as the type and location of the ligature, the bacteria species or their sub-products injected for the induction of the disease and the specie (strain), and age and weight of the animal used. These factors are generally associated with the objective of the study and the expected results.

More than 66% of studies chose the oral administration to evaluate the efficacy of the plant material. The topical administration was described by 26.6% of the studies and 6.9% treated animals through subcutaneous or intraperitoneal cavity injections. The route of administration is an important parameter to influence the efficacy of the material since it can interfere with the sufficient amount of substance available to promote the biological effects. As observed, different results were seen for alveolar bone loss of animals submitted to the treatment with an extract containing Catechin obtained from* Camellia sinensis*. Although the route of administration had not influenced the anti-inflammatory activity of the material, a significant reduction of bone loss was observed when given orally [23] and no difference was seen after its topical treatment [19].

Periodontal disease initiation and progression occur as a consequence of the host response to microorganisms of the dental biofilm [1]. Therefore, the antibacterial effect is an important factor in the periodontal therapy. Only 26.6% of the studies investigated the capacity of the material to present antibacterial activity. In the study developed by Barrella et al. [24] the organic extract obtained from* Ipomoea alba* showed significant* in vitro *activity against* Streptococcus mutans, S. sanguinis, *and* Enterococcus faecalis*. The commercial product Magnolol obtained from* Magnolia officinalis* exhibited intense inhibition of* Porphyromonas gingivalis* and* Aggregatibacter actinomycetemcomitans* growth in a dependent dose [11] and the aqueous extract from* Vaccinium macrocarpon* containing tannin and phenolic compounds inhibited the adhesion of* Fusobacterium nucleatum* and* Porphyromonas gingivalis* [15]. Study developed by Botelho et al. [25] and Botelho et al. [26] with* Lippia sidoides* observed the decrease of salivary bacterial levels and this event was followed by an increment in clinical scores of gingival bleeding. Except for the work published by Klausen et al. [8] which did not evaluate the alveolar bone loss, all the articles that described the identification of antibacterial activity also demonstrated that plant material treatment induced a significant reduction of alveolar bone loss.

The alveolar bone loss is one of the most important parameters evaluated in the induced periodontal disease. From 30 selected articles, 27 evaluated the ability of the plant material to promote a significant reduction of the bone loss. Carvacrol purified from* Lippia sidoides*, the extract containing Catechin obtained from* Camellia sinensis*, the essential oil of* Cordia verbenacea*, and the methanolic extract of* Hypericum perforatum* are examples of 77% of studies that observed a significant reduction of the bone loss [23, 26–28]. On the other hand, in 23% of studies such as andiroba oil from* Carapa guianensis*, as well as the mixture of dichloromethane and methanol extracts from* Ipomoea alba *and Longo Vital, the authors did not observe a significant effect [8, 21, 24]. Moreover, it was interesting to note that different studies using the same plant material have reached different results regarding alveolar bone loss. According to Yoshinaga et al. [29] the topical treatment of animals with the extract containing Catechin (*Camellia sinensis*) promoted a significant reduction of bone loss in periodontitis induced by* E. coli *(LPS). On the other hand, the same material did not present a significant reduction in the alveolar bone loss against periodontitis induced by injection of* E. coli* (LPS) and* S. griseus* (proteases) [19]. Different efficacy was also observed in studies developed with curcumin (*Curcuma longa*). The topical administration of curcumin did not reduce the alveolar bone loss [16] while its oral administration reduced this parameter significantly [10]. The methodologies used to evaluate the alveolar bone loss were histology (44.0%), stereomicroscopy (25.9%), microcomputed tomography (22.2%), atomic force microscopy (3.7%), and radiography (3.7%). Histology, stereomicroscopy, and microcomputed tomography are favorable methods once they yield precise information concerning the evaluation of periodontal tissue destruction.

It is noteworthy that the reduction of the inflammatory process may be associated with a reduced bone loss [12, 30, 31]. Because of this, a reduction of inflammatory process is usually investigated in periodontal disease. Taking the selected articles into account, 76.6% of them evaluated inflammatory parameters associated with induced periodontitis. The common strategies used to observe the ability of the plant material to inhibit the inflammatory process in periodontal tissue were the evaluation of migration of inflammatory cells (30%), measurement of proinflammatory cytokines (TNF-*α*: 30% and IL1-*β*: 13.3%) [2, 10, 12, 15, 19, 22, 23, 25, 32, 33], respectively, and dosage of myeloperoxidase (MPO) activity (20%) [11, 22, 25–27, 34]. Other molecular markers of inflammatory process, such as iNOS (13.0%) [11, 34], NF-*κ*B (8.7%) [27, 34], COX-2 (8.7%) [9, 11], and IL-10 (4.3%) [28], were also investigated. Although the presence of the anti-inflammatory effect reflects a possible efficacy against periodontitis, 13% of the authors who found anti-inflammatory properties did not observe a significant reduction of alveolar bone loss in the periodontitis assays. The anti-inflammatory activity was not analyzed in the 23.3% [6–8, 13, 20, 24, 35] of the studies. The andiroba oil (*Carapa guianensis*), the extract from* Camellia sinensis* (containing Catechin), and Curcumin (*Curcuma spp.*) were able to reduce the number of inflammatory cells in the gingival tissue. However, they were not effective to protect from destructive periodontal process [19, 21, 36]. According to the authors, these negative results may be related to the time delay to reach levels that are high enough for the biological effects of administered substances in the experimental models adopted. Further researches are necessary to observe how much time is appropriate to manage the substances for the recovery of bone loss.

Although there is a close relation between the bone reabsorption and the inflammatory response, we suggest that the negative results of some of substances analyzed should be attributed to the fact that they do not act in the osteoclastogenesis process. This information is supported by the personal observation conducted using sulphated polysaccharides recovered from red marine algae* Gracilaria caudata*. Our data revealed that the treatment of experimental animals with sulphated polysaccharides improved clinical and inflammatory parameters. However, no significant effect was observed in the reduction of alveolar bone loss (unpublished data).

It is important to mention that, although many plant substances may have deleterious effects to different animal organs [37], none of the evaluated studies has investigated the possible toxicological effects associated with the administration of the medicinal plants. In addition, several studies have documented that induced periodontitis is followed by significant changes of morphological structures and biochemical functions of different organs [38, 39]. Yet again, none of studies investigated the ability of these medicinal plants to prevent changes in organs promoted by a systemic action of the induced periodontitis.

Finally, 8 of the 30 selected articles conducted their experiments with a gel-based formulation containing the plant material investigated. In these experiments, the authors administered the gel topically, generally three times a day. The main idea behind these investigations is to reveal the further potential of the combined gel preparation to combat periodontal disease in closer than clinical situations. This was the outlook investigated in the studies published by [40, 41]. The authors evaluated the efficiency of a green tea Catechin gel as an adjunct on human periodontal therapy. In first work [40], the authors observed a significant reduction on pockets and inflammation during the 4 weeks of the clinical trial. In the second work [41], it was demonstrated that when used as an adjunct to periodontal treatment, green tea gel could provide benefit in reducing bleeding on probing and gingival inflammation at 1st and 3rd months of evaluation. What makes these studies even more relevant is the fact that the findings on complementary products for the treatment of human periodontitis still can be enhanced.

## 4. Conclusion

In conclusion, the selected studies presented a large diversity of experimental designs, concerning the type of induction, time of analysis, and methods used for the evaluation of alveolar bone loss, anti-inflammatory, and antibacterial activities. None of the studies evaluated the possible toxic effects associated with the administration of the material analyzed or their ability to prevent damages to organs caused by systemic effects of induced periodontitis. Gel-based formulations present an interesting strategy to treat periodontitis; however, further studies are necessary to clarify its usefulness in the clinical situation.

## Figures and Tables

**Table 1:** List of medicinal plants, experimental methods, and their biological effects on induced periodontitis.

Plant species	Plant material	Route of administration	Animal used (gender)	Type of induction/time of analysis	Antibacterial effect^1^	Bone loss/method^2^	Anti-inflammatory activity^3^	Reference
*Panax notoginseng *and *Rehmannia radix*	Mixture of extracts (9 : 1 weight)	Oral	*Rattus norvegicus* (male)	Injecting *E. coli* endotoxin (LPS)/28 days	Not evaluated	Reduced ABC/*µ*CT	Reduced the *in vitro* release of TNF-*α* from human monocytic cells and hGF cells	Almeida et al. [2]

*Ocimum sanctum*	Tulsi extract	Topical	*Rattus norvegicus* (male and female)	Silk ligature/9 days	Not evaluated	Not reduced ABC/Stereo	Presented anti-inflammatory activity in another experimental model	Hosadurga et al. [4]

*Scutellaria baicalensis*	Baicalin	Oral	*Rattus norvegicus* (male)	Nylon ligature/7 days	Not evaluated	Reduced ABC/histology	Not evaluated	Cai et al. [6]

*Rhizoma coptidis, Hydrastis canadensis, *and* Cortex phellodendri*	Berberine	Oral	*Rattus norvegicus* (male)	Silk ligature/8 days	Not evaluated	Reduced ABC/*µ*CT	Not evaluated	Tu et al. [7]

*Cucurbita pepo, Mentha piperita, Crataegus *spp.*, Rosmarinus officinalis, Capsicum annuum, *and* Achillea millefolium*	LongoVital®	Oral	*Rattus norvegicus* (male and female)	Injecting *A. viscosus* and *P. gingivalis*/63 days	Not evaluated	Reduced ABC/Radio	Not evaluated	Klausen et al. [8]

*Mangifera indica*	Mangiferin (Sigma-Aldrich Co.)	Oral	*Rattus norvegicus *(male)	Cotton ligature/1, 4, and 7 days	Not evaluated	Reduced ABC/histology	Inhibited COX-2 expression, the rolling, and adhesion of leukocytes in the periodontal tissue	Carvalho et al. [9]

*Curcuma longa *	Curcumin (Sigma-Aldrich Co.)	Gavage	*Rattus norvegicus (male)*	Nylon ligature/30 days	Not evaluated	Reduced ABC/histology	Reduced the expression of TNF-*α* in gingival tissues	Zhou et al. [10]

*Magnolia officinalis *	Magnolol (Sigma-Aldrich Co.)	Oral	*Rattus norvegicus *(male)	Silk ligature/9 days	*In vitro* activity against *P. gingivalis* and *A. actinomycetemcomitan*	Reduced ABC/*µ*CT	Inhibited neutrophil migration, MPO activity, and COX-2 and iNOS expression in gingival tissues	Lu et al. [11]

*Rhizoma drynariae *and *Rehmannia glutinosa*	Bu-Shen-Gu-Chi-Wan (*JiuZhiTang Pharmaceutical)*	Oral	*Rattus norvegicus* (female)	Injecting *P. gingivalis* and ligature/28 days	Not evaluated	Improved the mineral density of the bone/*µ*CT	Reduced levels of IL-1*β*, TNF-*α*, and inflammatory cell infiltration in the periodontal tissues	Yang et al. [12]

*Pinus pinaster*	Pycnogenol® (Tradepia Co.)	Oral	Balb/c (male)	Injecting *P. gingivalis*/34 days	Antibacterial activity against *P. gingivalis*	Reduced ABC/Stereo	Not evaluated	Sugimoto et al. [13]

*Vaccinium macrocarpon*	Aqueous extract containing tannin and phenolic compounds	Oral	*Mus musculus* (female)	Injecting *P. gingivalis* and *F. nucleatum*/42 days	Anti-adhesive properties against *P. gingivalis* and *F. nucleatum.* Increased the phagocytosis of *P. gingivalis*	Not evaluated	Reduced *in vivo* levels of TNF-*α*	Polak et al. [15]

*Curcuma longa*	Curcumin	Topical	*Rattus norvegicus*	Silk ligature/28 days	Not evaluated	Did not reduce ABC/Stereo	Exhibited anti-inflammatory activity in another experimental model	Hosadurga et al. [16]

*Camellia sinensis*	Extract containing Catechin	Topical	*Rattus norvegicus *(male)	Injecting *E. coli* (LPS) and *S. griseus* (proteases)/56 days	Not evaluated	Did not reduce ABC/histology	Reduced inflammatory cell infiltration and levels of TNF-*α* in the periodontal lesion	Maruyama et al. [19]

*Spatholobus suberectus*	Aqueous extract	Oral	*Mus musculus *(male)	Injecting *P. gingivalis* /42 days	*In vitro* antibacterial activity against *P. gingivalis*	Reduced ABC/Stereo	Not evaluated	Toyama et al. [20]

*Carapa guianensis*	Andiroba oil	Oral	*Rattus norvegicus* (male)	Cotton ligature/50 days	Not evaluated	Did not reduce ABC/histology	Reduced the quantity of inflammatory cells in histology	Carmona et al. [21]

*Protium heptaphyllum*	*α*-amyrin and *β*-amyrin	Oral	*Rattus norvegicus* (male)	Nylon ligature/1 day	Not evaluated	Not evaluated	Reduced gingival TNF-*α* levels and MPO activity	Holanda Pinto et al. [22]

*Camellia sinensis*	Extract containing Catechin	Oral	*Rattus norvegicus* (male)	Nylon ligature/7, 14, and 28 days	Not evaluated	Reduced ABC/histology	Reduced *in vivo* levels of TNF-*α*	Cho et al. [23]

*Ipomoea alba*	Mixture of dichloromethane and methanol extract	Topical	*Rattus norvegicus *(male)	Cotton ligature/11 days	*In vitro *antibacterial activity against *S. mutans *and *E. faecalis*	Did not reduce ABC/Stereo	Not evaluated	Barrella et al. [24]

*Lippia sidoides *and* Myracrodruon urundeuva*	Mixture of leaf essential oil and hydroalcoholic solution from bark	Topical	*Rattus norvegicus* (male)	Nylon ligature/11 days	Prevented the growth of oral microorganisms from gingival tissue	Reduced ABC/histology	Reduced MPO activity and inhibited TNF-*α* and IL-1*β* production in gingival tissue	Botelho et al. [25]

*Lippia sidoides*	Carvacrol	Topical	*Rattus norvegicus *(male)	Nylon ligature/11 days	Prevented the growth of oral microorganisms from gingival tissue	Reduced ABC/AFM	Reduced MPO activity in gingival tissue	Botelho et al. [26]

*Hypericum Perforatum*	Methanolic extract	Oral	*Rattus norvegicus *(male)	Silk ligature /8 days	Not evaluated	Reduced ABC/Stereo	Reduced inflammatory cell infiltration, vascular permeability, expression of NF-*κ*B, and MPO activity in gingivomucosal tissue	Paterniti et al. [27]

*Cordia verbenacea*	Essential oil	Topical	*Rattus norvegicus *(male)	Cotton ligature/11 days	Reduced *in vivo* frequency *of P. gingivalis* and *A. actinomycetemcomitans*	Reduced ABC/histology	Increment in the *in vivo* levels of IL-10	Pimentel et al. [28]

*Camellia sinensis*	Extract containing Catechin	Topical	*Rattus norvegicus* (male)	Injecting *E. coli *(LPS)/10 and 20 days	Not evaluated	Reduced ABC/histology	Reduced inflammatory cell infiltration	Yoshinaga et al. [29]

*Theobroma cacao*	Cocoa extract containing flavonoids	Oral	*Rattus norvegicus *(male)	Cotton ligature/28 days	Not evaluated	Reduced ABC/histology	Reduced/oxidized glutathione ratio and neutrophil infiltration	Tomofuji et al. [30]

*Mikania laevigata*	Ethanol extract	Subcutaneous	*Rattus norvegicus *(male)	Nylon ligature/30 days	Not evaluated	Reduced the furcation region/histology	Reduced neutrophil accumulation in the gingival tissue	Benatti et al. [31]

*Pimpinella anisum, Illicium verum, *and* Anethum foeniculum*	Anethole	Intraperitoneal	*Rattus norvegicus *(male)	Injecting *E. coli *(LPS)/10 days	Not evaluated	Not evaluated	Reduced serum levels of IL-1*β* and TNF-*α*	Moradi et al. [32]

*Curcuma *spp.	Modified curcumin	Gavage	*Rattus norvegicus (male)*	Injecting *E. coli* (LPS)/14 days	Not evaluated	Reduced ABC/Stereo, and *µ*CT	Reduced serum level of IL-1*β*	Elburki et al. [33]

*Syringa vulgaris*	Product of fermentation	Oral	*Rattus norvegicus* (male)	Silk ligature/8 days	Not evaluated	Reduced ABC/Stereo	Reduced NF-*κ*B and iNOS expression, MPO activity, and other inflammatory parameters in gingivomucosal tissue	Paola et al. [34]

*Ginkgo biloba*	*Ginkgo biloba *extract	Oral	*Rattus norvegicus *(male)	Silk ligature/11 days	Not evaluated	Reduced ABC/histology	Not evaluated	Sezer et al. [35]

*Curcuma *spp.	Curcumin	Gavage	*Rattus norvegicus (male)*	Cotton ligature/15 days	Not evaluated	Did not reduce ABC/*µ*CT	Reduced the inflammatory cell infiltrate to gingival tissue	Guimarães et al. [36]

^1^ *Porphyromonas gingivalis, Aggregatibacter actinomycetemcomitans, Streptococcus mutans, Streptococcus sanguinis, Enterococcus faecalis, *and* Fusobacterium nucleatum*. ^2^ABC: alveolar bone crest, *µ*CT: microcomputed tomography, AFM: atomic force microscopy, Stereo: stereomicroscopy, Radio_: _radiography. ^3^TNF-*α*: tumor necrosis factor *α*, hGF: hepatocyte growth factor, MPO: myeloperoxidase activity, IL-1*β*: interleukin-1 beta, IL-10: interleukin-10, NF-*κ*B: nuclear factor kappa-*β*, COX-2: ciclooxigenase-2, and iNOS: inducible nitric oxide synthase.
