# Corrigendum to “Biological Effects of Medicinal Plants on Induced Periodontitis: A Systematic Review”

**DOI:** 10.1155/2018/5656189

**Published:** 2018-02-27

**Authors:** Jefferson Soares de Oliveira, Moara e Silva Conceição Pinto, Lucas de Araújo de Bastos Santana, Antonione Santos Bezerra Pinto, David di Lenardo, Daniel Fernando Pereira Vasconcelos

**Affiliations:** ^1^Laboratory of Biology and Biochemistry Plants (BIOqPLANT), Federal University of Piaui, Parnaiba, PI, Brazil; ^2^Laboratory of Histological Analysis and Prepare (LAPHIS), Federal University of Piaui, Parnaiba, PI, Brazil; ^3^Department of Morphology, LABICONTE, Federal University of Ceara, Fortaleza, CE, Brazil

In the article titled “Biological Effects of Medicinal Plants on Induced Periodontitis: A Systematic Review” [1], it is stated in Table 1 that Hosadurga et al., cited as references 4 and 16 in the article, examined the anti-inflammatory action of 2% curcumin and *Ocimum sanctum* gel in a separate model. However, the authors of these articles informed us that they studied the gingival index, which is an indirect measure of inflammation in the gingival tissues. They also assessed the antimicrobial activity but did not publish the data, since they encountered a problem with the solubility and did not get a well-defined clear zone of inhibition. Corrected information about references [4] and [16] in Table 1 is as follows.

## Figures and Tables

**Table 1:** List of medicinal plants, experimental methods, and their biological effects on induced periodontitis.

Plant species	Plant material	Route of administration	Animal used (gender)	Type of induction/time of analysis		Antibacterial effect^1^	Bone loss/method^2^	Anti-inflammatory activity^3^	Reference	
*Panax notoginseng* and *Rehmannia radis*	Mixture of extracts (9 : 1 weight)	Oral	*Rattus norvegicus* (male)	Injecting *E. coli* endotoxin (LPS)/28 days		Not evaluated	Reduced ABC/*µ*CT	Reduced the *in vitro* release of TNF-*α* from human monocytic cells and hGF cells	Almeida et al. [2]	
*Ocimum sanctum*	Tulsi extract	Topical	*Rattus norvegicus* (male and female)	Silk ligature/9 days		Not evaluated	Not reduced ABC/Stereo	Presented anti-inflammatory activity in a separate model (paw edema)	Hosadurga et al. [4]	
*Scutellaria baicalensis*	Baicalin	Oral	*Rattus norvegicus* (male)	Nylon ligature/7 days		Not evaluated	Reduced ABC/histology	Not evaluated	Cai et al. [6]	
*Rhizoma coptidis*, *Hydrastis canadensis*, and *Cortex phellodendri*	Berberine	Oral	*Rattus norvegicus* (male)	Silk ligature/8 days		Not evaluated	Reduced ABC/*µ*CT	Not evaluated	Tu et al. [7]	
*Cucurbita pepo*, *Mentha piperita*, *Crataegus* spp., *Rosmarinus officinalis*, *Capsicum annuum*, *Achillea millefolium*	LongoVital®	Oral	*Rattus norvegicus* (male and female)	Injecting *A. viscosus* and *P. gingivalis*/63 days		Not evaluated	Reduced ABC/Radio	Not evaluated	Klausen et al. [8]	
*Mangifera indica*	Mangiferin (Sigma-Aldrich Co.)	Oral	*Rattus norvegicus* (male)	Cotton ligature/1, 4, 7 days		Not evaluated	Reduced ABC/histology	Inhibited COX-2 expression, the rolling, and adhesion of leukocytes in the periodontal tissue	Carvalho et al. [9]	
*Curcuma longa*	Curcumin (Sigma-Aldrich Co.)	Gavage	*Rattus norvegicus* (male)	Nylon ligature/30 days		Not evaluated	Reduced ABC/histology	Reduced the expression of TNF-*α* in gingival tissues	Zhou et al. [10]	
*Magnolia officinalis*	Magnolol (Sigma-Aldrich Co.)	Oral	*Rattus norvegicus* (male)	Silk ligature/9 days		*In vitro* activity against *P. gingivalis* and *A. actinomycetemcomitan*	Reduced ABC/*µ*CT	Inhibited neutrophil migration, MPO activity, and COX-2 and iNOS expression in gingival tissues	Lu et al. [11]	
*Rhizoma drynariae* and *Rehmannia glutinosa*	Bu-Shen-Gu-Chi-Wan (JiuZhiTang Pharmaceutical)	Oral	*Rattus norvegicus* (female)	Injecting *P. gingivalis* and ligature/28 days		Not evaluated	Improved the mineral density of the bone/*µ*CT	Reduced levels of IL-1*β*, TNF-*α*, and inflammatory cell infiltration in the periodontal tissues	Yang et al. [12]	
*Pinus pinaster*	Pycnogenol® (Tradepia Co.)	Oral	Balb/c (male)	Injecting *P. gingivalis*/34 days		Antibacterial activity against *P. gingivalis*	Reduced ABC/Stereo	Not evaluated	Sugimoto et al. [13]	
*Vaccinium macrocarpon*	Aqueous extract containing tannin and phenolic compounds	Oral	*Mus musculus* (female)	Injecting *P. gingivalis* and *F. nucleatum*/42 days		Antiadhesive properties against *P. gingivalis* and *F. nucleatum.* Increased the phagocytosis of *P. gingivalis*	Not evaluated	Reduced *in vivo* levels of TNF-*α*	Polak et al. [15]	
*Curcuma longa*	Curcumin	Topical	*Rattus norvegicus*	Silk ligature/28 days		Not evaluated	Did not reduce ABC/Stereo	Exhibited anti-inflammatory activity in a separate model (paw edema)	Hosadurga et al. [16]	
*Camellia sinensis*	Extract containing catechin	Topical	*Rattus norvegicus* (male)	Injecting *E. coli* (LPS) and *S. griseus* (proteases)/56 days		Not evaluated	Did not reduce ABC/histology	Reduced inflammatory cell infiltration and levels of TNF-*α* in the periodontal lesion	Maruyama et al. [19]	
*Spatholobus suberectus*	Aqueous extract	Oral	*Mus musculus* (male)	Injecting *P. gingivalis*/42 days		*In vitro* antibacterial activity against *P. gingivalis*	Reduced ABC/Stereo	Not evaluated	Toyama et al. [20]	
*Carapa guianensis*	Andiroba oil	Oral	*Rattus norvegicus* (male)	Cotton ligature/50 days		Not evaluated	Did not reduce ABC/histology	Reduced the quantity of inflammatory cells in histology	Carmona et al. [21]	
*Protium heptaphyllum*	*α-*Amyrin and *β*-amyrin	Oral	*Rattus norvegicus* (male)	Nylon ligature/1 day		Not evaluated	Not evaluated	Reduced gingival TNF-*α* levels and MPO activity	Holanda Pinto et al. [22]	
*Camellia sinensis*	Extract containing catechin	Oral	*Rattus norvegicus* (male)	Nylon ligature/7, 14, 28 days		Not evaluated	Reduced ABC/histology	Reduced *in vivo* levels of TNF-*α*	Cho et al. [23]	
*Ipomoea alba*	Mixture of dichloromethane and methanol extract	Topical	*Rattus norvegicus* (male)	Cotton ligature/11 days		*In vitro* antibacterial activity against *S. mutans*, *S.* and *E. faecalis*	Did not reduce ABC/Stereo	Not evaluated	Barrella et al. [24]	
*Lippia sidoides* and *Myracroduon urundeuva*	Mixture of leaf essential oil and hydroalcoholic solution from bark	Topical	*Rattus norvegicus* (male)	Nylon ligature/11 days		Prevented the growth of oral microorganisms from gingival tissue	Reduced ABC/histology	Reduced MPO activity and inhibited TNF-*α* and IL-1*β* production in gingival tissue	Botelho et al. [25]	
*Lippia sidoides*	Carvacrol	Topical	*Rattus norvegicus* (male)	Nylon ligature/11 days		Prevented the growth of oral microorganisms from gingival tissue	Reduced ABC/AFM	Reduced MPO activity in gingival tissue	Botelho et al. [26]	
*Hypericum perforatum*	Methanolic extract	Oral	*Rattus norvegicus* (male)	Silk ligature/8 days	Not evaluated		Reduced ABC/Stereo	Reduced inflammatory cell infiltration, vascular permeability, expression of NF-*κ*B and MPO activity in gingivomucosal tissue		Paterniti et al. [27]
*Cordia verbenaea*	Essential oil	Topical	*Rattus norvegicus* (male)	Cotton ligature/11 days	Reduced in vivo frequency of *P. gingivalis* and *A. actinomycetemcomitans*		Reduced ABC/histology	Increment in the in vivo levels of IL-10		Pimentel et al. [28]
*Camellia sinensis*	Extract containing catechin	Topical	*Rattus norvegicus* (male)	Injecting *E. coli* (LPS)/10 and 20 days	Not evaluated		Reduced ABC/histology	Reduced inflammatory cell infiltration		Yoshinaga et al. [29]
*Theobroma cacao*	Cocoa extract containing flavonoids	Oral	*Rattus norvegicus* (male)	Cotton ligature/28 days	Not evaluated		Reduced ABC/histology	Reduced/oxidized glutathione ratio and neutrophil infiltration		Tomofuji et al. [30]
*Mikania laevigata*	Ethanol extract	Subcutaneous	*Rattus norvegicus* (male)	Nylon ligature/30 days	Not evaluated		Reduced the furcation region/histology	Reduced neutrophil accumulation in the gingival tissue		Benatti et al. [31]
*Pimpinella anisum*, *Illicium verum*, *Anethum foeniculum*	Anethole	Intraperitoneal	*Rattus norvegicus* (male)	Injecting *E. coli* (LPS)/10 days	Not evaluated		Not evaluated	Reduced serum levels of IL-1*β* and TNF-*α*		Moradi et al. [32]
*Curcuma* spp.	Modified curcumin	Gavage	*Rattus norvegicus* (male)	Injecting *E. coli* (LPS)/14 days	Not evaluated		Reduced ABC/Stereo, and *µ*CT	Reduced serum level of IL-1*β*		Elburki et al. [33]
*Syringa vulgaris*	Product of fermentation	Oral	*Rattus norvegicus* (male)	Silk ligature/8 days	Not evaluated		Reduced ABC/Stereo	Reduced NF-*κ*B and iNOS expression, MPO activity, and other inflammatory parameters in gingivomucosal tissue		Paola et al. [34]
*Ginkgo biloba*	Ginkgo biloba extract	Oral	*Rattus norvegicus* (male)	Silk ligature/11 days	Not evaluated		Reduced ABC/histology	Not evaluated		Sezer et al. [35]
*Curcuma* spp.	Curcumin	Gavage	*Rattus norvegicus* (male)	Cotton ligature/15 days	Not evaluated		Did not reduce ABC/*µ*CT	Reduced the inflammatory cell infiltrate to gingival tissue		Guimarães et al. [36]

^1^ *Porphyromonas gingivalis*, *Aggregatibacter actinomycetemcomitan*, *Streptococcus mutans*, *Streptococcus sanguinis*, *Enterococcus faecalis*, and *Fusobacterium nucleatum*. ^2^ABC: alveolar bone crest, *µ*CT: microcomputed tomography, AFM: atomic force microscopy, Stereo: stereomicroscopy, Radio: radiography. ^3^TNF-*α*: tumor necrosis factor *α*, hGF: hepatocyte growth factor, MPO: myeloperoxidase activity, IL-1*β*: interleukin-1 beta, IL-10: interleukin-10, NF-*κ*B: nuclear factor kappa-*β*, COX-2: cyclooxygenase-2, and iNOS: inducible nitric oxide synthase.
